# Preperitoneal analgesia under direct visualization: laparoscopic-guided deep rectus sheath block improves postoperative pain control after laparoscopic cholecystectomy: a randomized controlled trial

**DOI:** 10.1007/s00464-026-12973-5

**Published:** 2026-06-16

**Authors:** Ali Sait Kavakli, Mehmet Abdussamet Bozkurt, Musa Diri, Taylan Sahin, Umit Koc, Zeynep Su Kardelen Ipek, Ares Cekem

**Affiliations:** 1 Department of Anesthesiology and Reanimation, Faculty of Medicine, Istinye University, Istanbul, Turkey; 2 Department of General Surgery, Faculty of Medicine, Istinye University, Istanbul, Turkey; 3 Istinye University Hospital, Asik Veysel Mh. Suleyman Demirel Cd. No:1, Esenyurt, Istanbul, Turkey

**Keywords:** Deep rectus sheath block, Preperitoneal block, Laparoscopic cholecystectomy, Regional anesthesia, Postoperative analgesia

## Abstract

**Background:**

Conventional abdominal wall blocks primarily target somatic afferent nerves and may therefore provide limited relief for the peritoneal component of postoperative pain. This study aimed to investigate whether a laparoscopic-guided deep rectus sheath (preperitoneal) block (RSB) could reduce postoperative opioid consumption and improve postoperative pain in patients undergoing laparoscopic cholecystectomy.

**Methods:**

Patients were randomly assigned to either the control group or the deep RSB group. In the deep RSB group, the block was performed intraoperatively under direct laparoscopic visualization. No block was performed in the control group. In the deep RSB group, postoperative ultrasound assessment was used to verify bilateral local anesthetic localization. Postoperatively, all patients were connected to an intravenous patient-controlled analgesia device containing morphine. The primary outcome was cumulative morphine consumption within the first 24 postoperative hours.

**Results:**

Cumulative 24-h morphine consumption was significantly lower in the deep RSB group, with a median difference of − 9 mg (95% CI − 11 to − 6; *p* = 0.0001). Pain scores at rest and during movement were significantly lower in the deep RSB group at 2, 6, and 12 h postoperatively, with no significant difference observed at 24 h. Rescue analgesia requirement and incidence of postoperative nausea and vomiting were similar between groups. Quality-of-recovery scores at 24 h were significantly higher in the deep RSB group.

**Conclusions:**

Laparoscopic-guided deep RSB significantly reduced postoperative opioid consumption and improved early postoperative pain control after laparoscopic cholecystectomy. Targeting the preperitoneal plane under direct laparoscopic visualization may enhance early multimodal analgesia in these patients.

**Trial registry:**

ClinicalTrials.gov identifier: NCT06976320.

Postoperative pain following laparoscopic cholecystectomy is multifactorial, comprising somatic pain from trocar incisions, parietal peritoneal irritation, and visceral pain related to gallbladder bed dissection and diaphragmatic irritation secondary to pneumoperitoneum [1]. Although overall pain intensity is lower than after open abdominal surgery, inadequate early analgesia may delay mobilization, impair recovery quality, and increase opioid requirements during the immediate postoperative period [2, 3].

Multimodal analgesia is widely recommended for laparoscopic cholecystectomy and typically includes paracetamol, non-steroidal anti-inflammatory drugs, port-site infiltration, and selective opioid administration [4]. Regional anesthesia techniques targeting the anterior abdominal wall, such as the transversus abdominis plane (TAP) block and rectus sheath block (RSB), have been investigated to reduce postoperative pain and opioid consumption. However, these techniques predominantly target somatic afferents of the abdominal wall and may have limited influence on the parietal peritoneal component of pain [5]. Consequently, evidence supporting the routine use of classical RSB in laparoscopic cholecystectomy remains inconsistent, and current procedure-specific recommendations do not uniformly endorse its application [5, 6].

The parietal peritoneum is highly sensitive to pressure, incision, and inflammatory stimuli due to its somatic innervation, and therefore contributes substantially to early postoperative pain after laparoscopic surgery [7]. Conventional abdominal wall blocks may provide limited coverage of this component because they primarily target somatic afferents of the anterior abdominal wall [5]. Accordingly, targeting the preperitoneal plane beneath the posterior rectus sheath has been proposed as a strategy to extend analgesic coverage beyond the abdominal wall. Preperitoneal local anesthetic infiltration was initially described in laparoscopic hernia repair [8], and more recently the deep RSB has been introduced as an ultrasound-guided technique targeting this plane [9]. However, evidence supporting deep RSB or preperitoneal approaches remains limited and is largely based on case reports or small case series [10–14].

The present study aimed to evaluate whether laparoscopically guided deep rectus sheath (preperitoneal) block reduces postoperative opioid consumption and improves early postoperative pain control in patients undergoing elective laparoscopic cholecystectomy. We hypothesized that preperitoneal deposition of local anesthetic under direct laparoscopic visualization would result in a clinically meaningful reduction in 24-h morphine consumption compared with standard multimodal analgesia alone.

## Methods

### Study design

This prospective, parallel-group, two-arm randomized controlled trial was conducted in adult patients undergoing elective laparoscopic cholecystectomy. The study was approved by the Istinye University Faculty of Medicine Ethics Committee, Istanbul, Turkey (No. 25/98), and was registered at ClinicalTrials.gov (Identifier: NCT06976320) on May 09, 2025, prior to patient enrollment. The trial was conducted in accordance with the principles of the Declaration of Helsinki, and written informed consent was obtained from all participants. The study was reported in accordance with the Consolidated Standards of Reporting Trials (CONSORT) guidelines.

### Participants

The study was conducted at Istinye University Hospital, Istanbul, Turkey between September 2025 and February 2026, and adult patients scheduled for elective laparoscopic cholecystectomy were screened for eligibility. Patients aged 18 years or older with an American Society of Anesthesiologists (ASA) physical status of I or II were included in the study. Exclusion criteria included a known allergy to local anesthetics, a history of chronic opioid use or chronic pain, coagulopathy or ongoing anticoagulant therapy, significant hepatic or renal dysfunction, and any condition impairing communication or cooperation that could interfere with reliable pain assessment or the use of patient-controlled analgesia (PCA).

### Randomization and blinding

Before surgery, all patients were visited on the evening prior to the operation by an anesthesiologist who was not involved in intraoperative management or postoperative assessments. During this visit, patients received standardized information about the study protocol, and those who agreed to participate were instructed on the use of the Numerical Rating Scale (NRS) for pain assessment and the PCA device. Eligible patients were randomly assigned in a 1:1 ratio to either the laparoscopic-guided deep RSB group or the control group. Randomization was performed using a computer-generated random sequence by a member of the research team who was not involved in patient care, perioperative management, postoperative assessments, or data analysis. Group allocations were placed in sequentially numbered, opaque, sealed envelopes to ensure allocation concealment, which were opened immediately before surgery by the surgeon responsible for performing the block.

All surgical procedures were performed by the same surgical team to ensure procedural consistency. In the block group, all laparoscopic-guided deep RSBs were performed by the same surgeon. The surgical team performing the block was not involved in postoperative patient evaluations, pain assessments, or data collection. Due to the nature of the intervention, blinding of the surgeon performing the block was not feasible. However, all postoperative outcome assessors, including nursing staff responsible for pain assessment and data recording, were blinded to group allocation. Data analysts remained blinded to group allocation until the primary analysis was completed.

### Anesthesia management

All patients received a standardized general anesthesia protocol. Standard monitoring was applied upon arrival in the operating room, including electrocardiography, noninvasive blood pressure measurement, and pulse oximetry. After preoxygenation, anesthesia was induced with intravenous propofol (2–2.5 mg/kg), fentanyl (1–2 μg/kg), and rocuronium (0.6 mg/kg) to facilitate tracheal intubation. Following endotracheal intubation, anesthesia was maintained with sevoflurane in an oxygen–air mixture, and additional doses of rocuronium were administered as required to maintain adequate neuromuscular blockade. Mechanical ventilation was provided in volume-controlled mode with a tidal volume of 6–8 mL/kg of predicted body weight, a positive end-expiratory pressure of 5 cmH_2_O, and a respiratory rate adjusted to maintain end-tidal carbon dioxide between 35 and 40 mmHg. Pneumoperitoneum pressure was maintained at a maximum of 12 mmHg throughout the procedure.

Intraoperative analgesia was provided using intravenous fentanyl administered in incremental boluses. Additional fentanyl doses were administered when mean arterial pressure or heart rate increased by more than 20% compared with baseline values, in accordance with the standardized institutional protocol. Approximately 15 min before the end of surgery, all patients received intravenous paracetamol (1 g) and tramadol (100 mg) as part of a multimodal analgesic regimen. Intravenous dexamethasone (4 mg) was administered after induction of anesthesia, and ondansetron (4 mg) was administered intravenously 10 min before the end of surgery for prophylaxis against postoperative nausea and vomiting. At the end of surgery, neuromuscular blockade was reversed using sugammadex, and tracheal extubation was performed after confirmation of adequate spontaneous ventilation, recovery of neuromuscular function, and hemodynamic stability. All patients were subsequently transferred to the post-anesthesia care unit (PACU) for postoperative monitoring and pain management according to the study protocol. After at least 30 min of observation, patients were transferred to the surgical ward.

### Laparoscopic-guided deep rectus sheath block technique

In patients allocated to the block group, a laparoscopic-guided deep RSB was performed intraoperatively after the end of the surgical procedure and before emergence from anesthesia. The block was carried out under direct laparoscopic visualization by the same surgeon in all cases to ensure technical consistency.

**Fig. 1:**
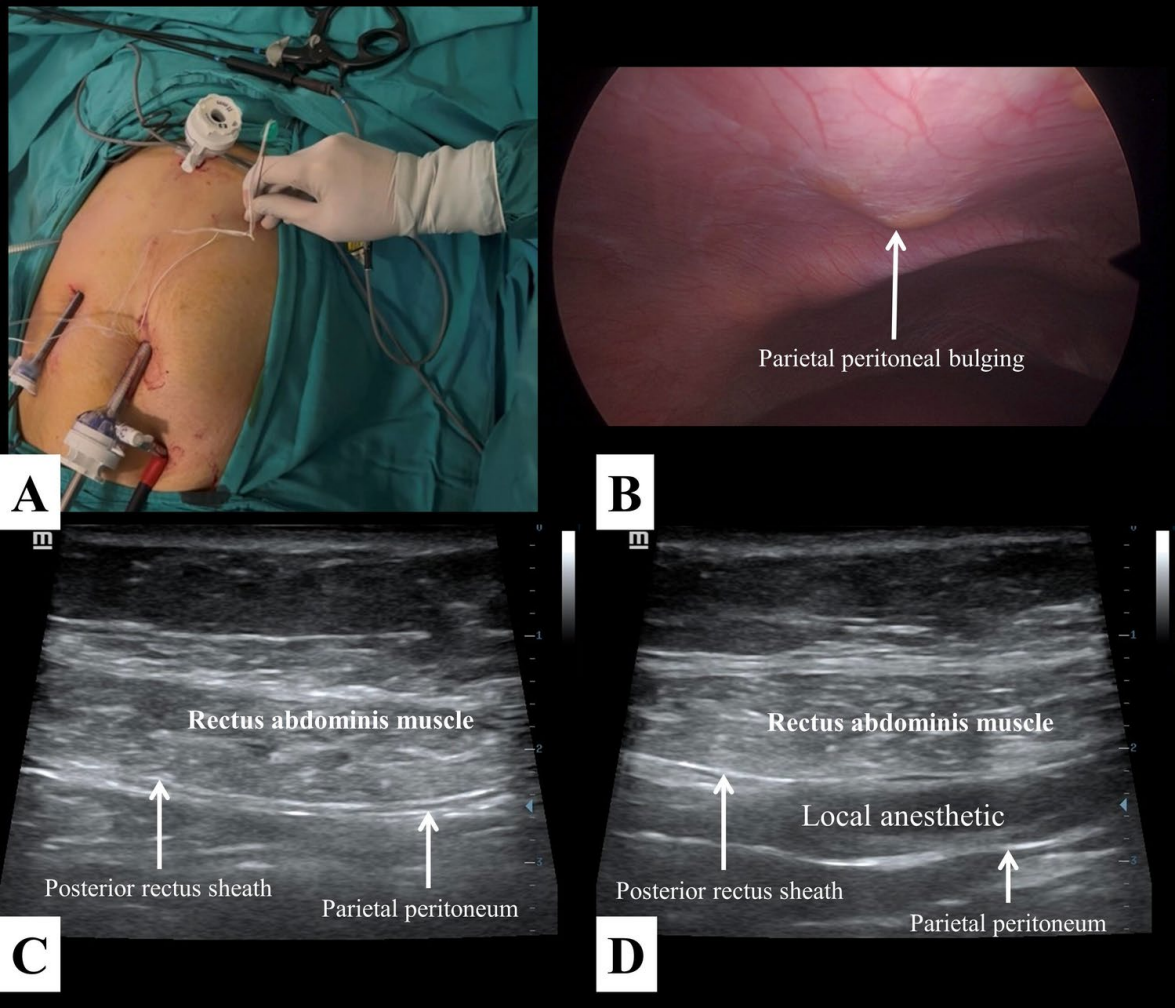
Technique and imaging of laparoscopic-guided deep rectus sheath block. **A** Intraoperative demonstration of laparoscopic-guided deep rectus sheath block during laparoscopic cholecystectomy. **B** Laparoscopic view demonstrating convex bulging of the parietal peritoneum following preperitoneal injection of local anesthetic. **C** Ultrasound image of the anterior abdominal wall before injection showing the rectus abdominis muscle, posterior rectus sheath, and parietal peritoneum. **D** Ultrasound image after injection demonstrating spread of local anesthetic in the preperitoneal plane deep to the posterior rectus sheath

The target injection plane was defined as the preperitoneal space between the posterior rectus sheath and the parietal peritoneum, which is anatomically distinct from the plane targeted in the classical RSB [9]. Under direct laparoscopic visualization from the umbilical port during pneumoperitoneum, a 22-gauge 100-mm needle (Stimuplex® Ultra 360, B. Braun) was inserted percutaneously at the lateral border of the rectus abdominis muscle, with continuous laparoscopic visualization during advancement (Fig. 1A). After passing through the rectus muscle and penetrating the posterior rectus sheath, the needle tip was advanced into the potential preperitoneal plane. Correct needle positioning was confirmed laparoscopically by visualization of the needle tip beneath the posterior rectus sheath with indentation of the intact parietal peritoneum. Incremental injection of local anesthetic produced hydrodissection and expansion of this plane, which resulted in visible convex displacement of the parietal peritoneum without evidence of intraperitoneal spread (Fig. 1B). The block was performed bilaterally using 20 mL of 0.25% bupivacaine on each side, resulting in a total volume of 40 mL.

No additional local anesthetic infiltration or regional block was performed in the control group. Apart from the block intervention, all perioperative and postoperative management protocols were identical between the two groups.

### Postoperative ultrasound confirmation of block placement

A bilateral ultrasound examination was performed in patients in the deep RSB group both immediately after induction of anesthesia (baseline assessment) and at the end of the surgical procedure following block administration to verify the distribution of local anesthetic at both injection sites. The baseline examination was performed to document the pre-injection anatomy and to allow comparison with the postoperative ultrasound recordings. Ultrasound assessment was conducted solely for verification purposes and was not incorporated into postoperative outcome evaluation. The examination was performed using a Mindray Z60 diagnostic ultrasound system (Mindray Bio-Medical Electronics Co., Shenzhen, China) and a 5–10 MHz linear transducer (Mindray 7L4P, Shenzhen, China). All ultrasound scans were obtained by a single anesthesiologist experienced in ultrasound-guided regional anesthesia. For each patient, both sides of the anterior abdominal wall were examined separately, and short video clips of the ultrasound examination were recorded and stored for subsequent blinded evaluation. The rectus abdominis muscle, posterior rectus sheath, and peritoneal interface were systematically identified, and the anatomical relationship between these structures was assessed (Fig. 1C, D).

Correct preperitoneal/deep rectus sheath localization was defined by visualization of a hypoechoic or anechoic fluid collection situated deep to the posterior rectus sheath and superficial to the peritoneal line, with preservation of the peritoneal interface and absence of predominant intramuscular or subcutaneous spread. Localization was considered incorrect when fluid distribution was confined within the rectus abdominis muscle, remained superficial to the posterior rectus sheath without extension into the preperitoneal plane, or accumulated within the subcutaneous layer. Examinations were classified as non-evaluable when the relevant anatomical planes could not be reliably distinguished due to inadequate visualization, acoustic artifact, or inability to confidently localize residual fluid within a defined fascial compartment.

The recorded ultrasound clips were subsequently reviewed independently by two anesthesiologists experienced in regional anesthesia who were not involved in postoperative outcome assessments and were blinded to clinical data. Each reviewer performed the evaluation separately and was unaware of the other reviewer’s classification. Image interpretation was conducted in a standardized manner to prevent interaction or influence between assessors. Each block site was categorized as correct, incorrect, or non-evaluable. Concordant classifications were accepted as final. In cases of discordant classification, the ultrasound clips were reviewed by a third blinded senior anesthesiologist, and the final classification was determined by majority agreement.

For patient-level inclusion in the final analyses, bilateral majority-confirmed correct localization was required. Only patients with majority-confirmed correct localization on both sides were included. Patients with incorrect or non-evaluable localization on either side were excluded from the final analyses.

### Postoperative analgesia protocol

Postoperative pain management was standardized for all patients. Pain intensity was assessed using the Numerical Rating Scale (NRS), where 0 represented no pain and 10 indicated the worst imaginable pain. In the PACU, pain assessments were performed by a protocol-trained nurse who was blinded to group allocation. Patients presenting with an NRS score > 4 in the PACU received an initial intravenous bolus of morphine (2 mg). Additional 2-mg doses were administered at 10-min intervals as required until the NRS score decreased to ≤ 4. Before discharge from the PACU, all patients were connected to an intravenous PCA device. The PCA device was programmed with a morphine concentration of 0.5 mg/mL, allowing patient-administered bolus doses of 1 mg with a lockout interval of 8 min and a maximum dose limit of 6 mg per hour. No background infusion was used. Patients were instructed to activate the device only when their NRS score exceeded 4.

Following the arrival at the general surgery ward, all patients received scheduled intravenous paracetamol at a dose of 1 g three times daily as part of a multimodal analgesic regimen. Intravenous diclofenac (50 mg) was administered as rescue analgesia when pain control was inadequate despite PCA use, in accordance with the predefined study protocol. Postoperative NRS scores at rest and during movement were recorded at predefined time points, including in the PACU and at 2, 6, 12, and 24 h postoperatively.

### Outcome measures

The primary outcome of the study was the total cumulative morphine consumption during the first 24 h postoperatively. Secondary outcomes included the postoperative pain intensity assessed using NRS scores in the PACU and at 2, 6, 12, and 24 h postoperatively, the requirement for rescue analgesia within the first 24 h, total intraoperative fentanyl consumption, recovery quality at 24 h postoperatively assessed using the Quality of Recovery-15 (QoR-15) questionnaire [15, 16], the incidence of postoperative nausea and vomiting, and block-related complications.

### Statistical analyses

The primary analysis was performed according to a modified intention-to-treat principle including all randomized patients for whom the primary outcome (24-h morphine consumption) could be reliably measured. Patients in whom the primary outcome could not be assessed due to technical reasons, such as malfunction of the PCA device or inability to confirm correct injectate localization on postoperative ultrasound examination, were excluded from the final analysis.

Sample size estimation was based on the primary outcome of cumulative morphine consumption within the first 24 h postoperatively and was performed using G*Power software (version 3.1.9.2). In a preliminary cohort of patients undergoing laparoscopic cholecystectomy without a block, the mean ± SD 24-h morphine consumption was 13.5 ± 6.8 mg. Based on previous randomized studies of classical RSB, it was assumed that a reduction of at least 50% in 24-h morphine consumption in the block group compared with the control group would be clinically relevant [17], corresponding to an absolute difference exceeding the reported minimal important difference for postoperative opioid consumption within the first 24 postoperative hours [18]. Assuming equal standard deviations between groups, a two-sided significance level of 0.05, and a power of 90%, the required sample size was calculated to be 23 patients per group. To compensate for potential exclusions and incomplete follow-up, a total sample size of 60 patients was planned, with 30 patients allocated to each group.

Statistical analyses were performed using SPSS software (version 24.0; SPSS Inc., Chicago, IL, USA). Continuous variables were assessed for normality using the Shapiro–Wilk test. Normally distributed data were presented as mean ± standard deviation and were compared using the independent samples *t* test, whereas non-normally distributed data were presented as median with interquartile range (IQR) and were compared using the Mann–Whitney *U* test. Categorical variables were presented as counts and percentages and were compared using the chi-square test or Fisher’s exact test, as appropriate. For categorical outcomes, relative risks (RRs) with 95% confidence intervals (CIs) were calculated to quantify the magnitude of between-group differences. For non-normally distributed continuous outcomes, median differences with 95% CIs were estimated using the Hodges–Lehmann estimator to provide an estimate of the median difference as a measure of effect size. Interobserver agreement for ultrasound classification was assessed using Cohen’s kappa coefficient, and the percentage of observed agreement was also reported. All statistical tests were two-sided, and a *p* value < 0.05 was considered statistically significant.

## Results

**Fig. 2:**
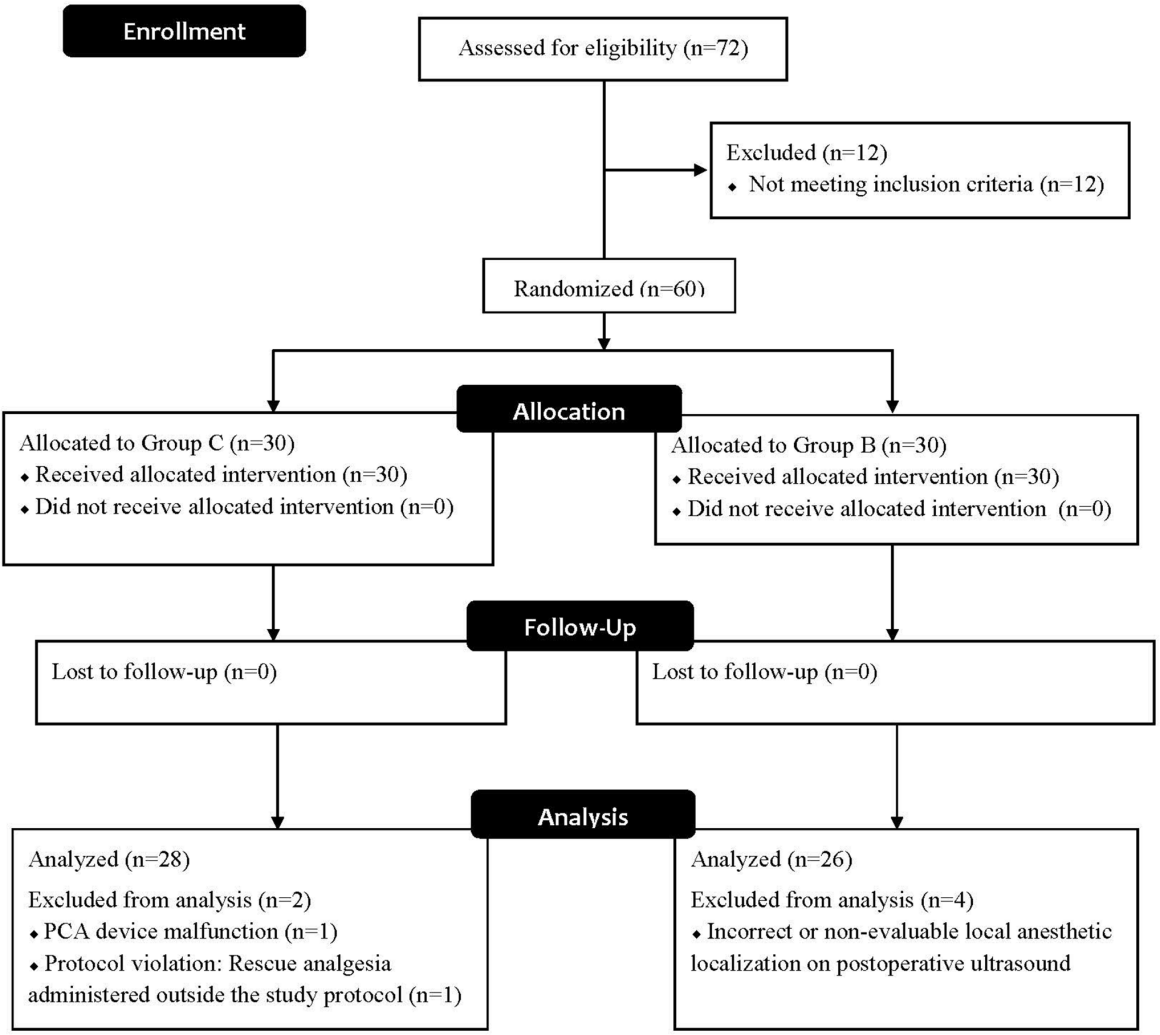
CONSORT flow diagram

A total of 72 patients were assessed for eligibility. Of these, 12 patients were excluded because they did not meet the study inclusion criteria. The remaining 60 patients were randomized to either the control group or the deep RSB group. After randomization, six patients were excluded from the final analysis. In the control group, two patients were excluded due to PCA device malfunction in one patient and administration of rescue analgesics outside the predefined study protocol in the other. In the block group, four patients were excluded because correct injectate localization could not be confirmed bilaterally on postoperative ultrasound assessment according to the predefined criteria. Consequently, the final analysis included 28 patients in the Control Group and 26 patients in the deep RSB Group (Fig. 2).

Postoperative bilateral ultrasound examination was completed in all 30 patients in the block group, corresponding to 60 block sites. At the block level, the first assessor classified 52 sites as correct, 5 as incorrect, and 3 as non-evaluable, whereas the second assessor classified 53 sites as correct, 4 as incorrect, and 3 as non-evaluable. Observed agreement between the two assessors was 88.3% (53/60 sites). Interobserver agreement was moderate (Cohen’s *κ* = 0.49, *p* < 0.001). Discordant classification occurred in 7 of 60 block sites (11.7%). These sites were subsequently reviewed by a third blinded senior anesthesiologist, and final classification was determined by majority agreement. Based on the final block-level classification, bilateral correct localization was confirmed in 26 of 30 patients (86.7%), whereas 4 patients (13.3%) had incorrect or non-evaluable localization on at least one side.

Baseline demographic and perioperative characteristics, including age, sex, body mass index, ASA physical status, duration of surgery, and duration of anesthesia, were similar between groups (Table 1).

**Table 1:** Demographic and perioperative characteristics

	Control group (*n* = 28)	Deep RSB group (*n* = 26)	*P* value
Age, years	46.1 ± 12.5	43.3 ± 8.4	0.341
Sex, female/male	12/16 (42.9/57.1)	10/16 (38.5/61.5)	0.743
BMI, kg/m^2^	27.3 ± 4.6	27.8 ± 4.4	0.724
ASA physical status, I/II	6/22 (21.4/78.6)	8/18 (30.8/69.2)	0.434
Baseline QoR-15 score	145.5 (143–146)[138–148])	143 (141–145 [132–148])	0.073
Duration of surgery, min	48 ± 6	45 ± 8	0.114
Duration of anesthesia, min	60 ± 7	57 ± 6	0.099

Values are presented as mean ± SD, median (IQR [range]) or number of patients (%)

*BMI* body mass index, *ASA* American Society of Anesthesiologists, *QoR* quality of recovery

Total cumulative morphine consumption over the first 24 postoperative hours was significantly lower in the deep RSB group compared with the control group. At 24 h, morphine consumption was reduced by a median of 9 mg in the block group, corresponding to a median difference of − 9 mg (95% CI − 11 to − 6; *p* = 0.0001). Significant reductions in cumulative morphine consumption were also observed at 2, 6, and 12 h postoperatively (all *p* = 0.0001). Morphine consumption in the PACU did not differ significantly between groups, with a median (IQR [range]) of 2.5 (1.5 to 3.5 [0–4]) mg in the control group and 2 (0 to 3 [0–4]) mg in the deep RSB group (*p* = 0.074) (Table 2). In the PACU, 25 patients in the control group and 18 patients in the deep RSB group required rescue morphine (*p* = 0.082). In an exploratory descriptive analysis, the four excluded patients in the deep RSB group had a 24-h morphine consumption of 8.5 mg (5 to 14.25 [4–16]), which remained lower than the median value observed in the control group.

**Table 2:** Cumulative morphine consumption

	Control group (*n* = 28)	Deep RSB group (*n* = 26)	Difference (95% CI)	*P* value
PACU	2.5 (1.5 to 3.5 [0–4])	2 (0 to 3 [0–4])	−1 (−1 to 0)	0.074
2 h	6 (5 to 8 [2–10])	2.5 (2 to 4 [2–8])	−3 (−4 to −2)	0.0001
6 h	10 (8 to 13.5 [3–16])	4 (3 to 6 [2–13])	−6 (−8 to −4)	0.0001
12 h	13 (10 to 16.5 [5–21])	5 (4 to 7 [2–17])	−8 (−10 to −5)	0.0001
24 h	15 (12.5 to 19 [7–24])	6.5 (5 to 8 [3–20])	−9 (−11 to −6)	0.0001

Values are presented as median (IQR [range]) and median difference with 95% CI

**Fig. 3:**
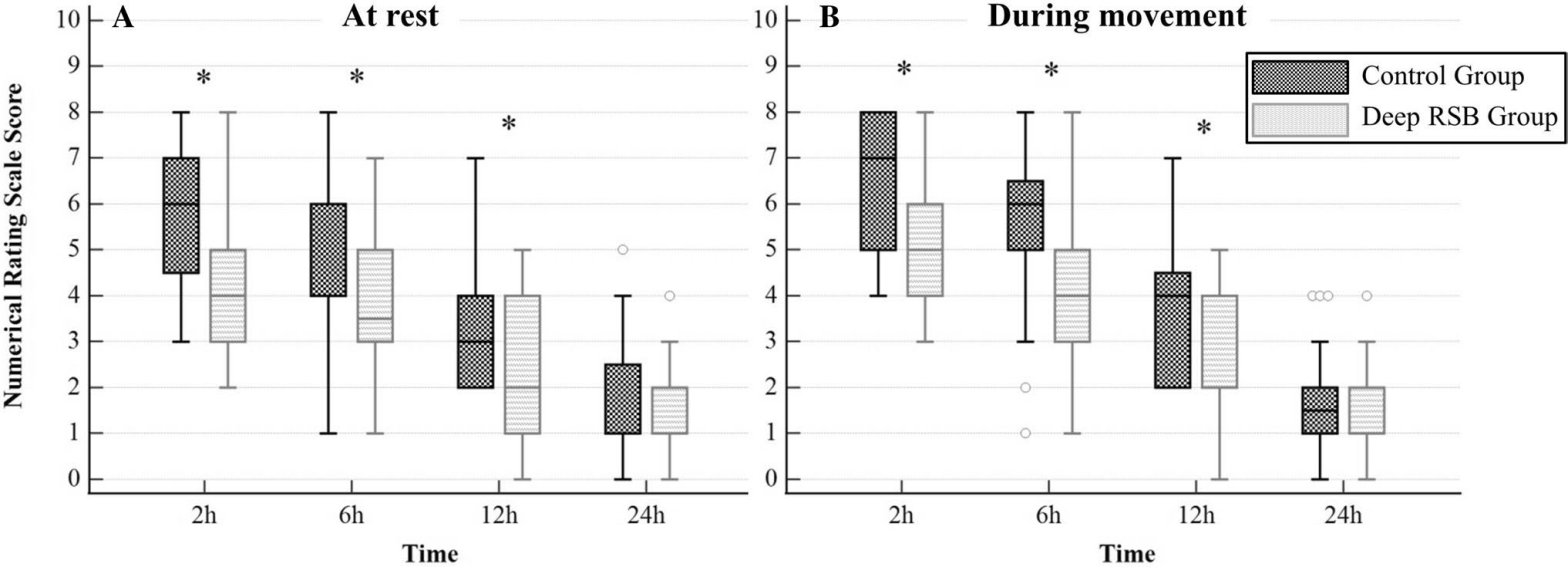
Postoperative NRS pain scores in the control and deep RSB groups. **A** Pain scores at rest. **B** Pain scores during movement. Box plots represent the median and interquartile range (IQR); whiskers indicate minimum and maximum values

Postoperative pain scores assessed using the NRS at rest and during movement differed between groups at several postoperative time points. At rest, NRS scores were significantly lower in the block group at 2, 6, and 12 h postoperatively, while no significant difference was observed at 24 h (*p* = 0.0001, 0.003, 0.030, and 0.387, respectively). Similarly, during movement, NRS scores were significantly lower in the deep RSB group at 2, 6, and 12 h, with no significant difference at 24 h (*p* = 0.001, 0.004, 0.036, and 0.448, respectively). No significant differences in NRS scores were observed between groups in the PACU (*p* = 0.337). NRS values at rest and during movement are presented in Fig. 3.

Rescue analgesia was required within the first 24 postoperative hours in 10 patients (35.7%) in the control group and 6 patients (23.1%) in the deep RSB group. There was no statistically significant difference between the two groups in terms of rescue analgesia requirement (*p* = 0.320). At 24 h postoperatively, QoR-15 scores were significantly higher in the block group compared with the control group (*p* = 0.0001). There was no statistically significant difference between the two groups in the incidence of PONV (*p* = 0.390). No block-related complications, such as peritoneal perforation, visceral injury, bleeding, or local anesthetic systemic toxicity, were observed intraoperatively or during the first 24 postoperative hours (Table 3).

**Table 3:** Secondary outcomes

	Control group (*n* = 28)	Deep RSB group (*n* = 26)	Effect size (95% CI)	*P* value
Intraoperative fentanyl requirement, µg	150 (100–150 [100–200])	150 (100–150 [100–200])	0 (0 to 50)	0.509
Postoperative QoR-15 scores	131 (124–135 [117–140])	140 (135–142 [130–148])	8 (5 to 12)	0.0001
Number of patients requiring morphine in the PACU	25 (89.3)	18 (69.2)	0.78 (0.58 to 1.03)	0.082
Number of patients requiring postoperative rescue analgesia	10 (35.7)	6 (23.1)	0.65 (0.27 to 1.53)	0.320
PONV	7 (25)	4 (15.4)	0.62 (0.20 to 1.86)	0.390

Values are presented as median (IQR [range]) or number of patients (%). Effect size is presented as median difference for continuous variables and relative risk for categorical variables, both with 95% confidence intervals

*QoR* quality of recovery, *PACU* post-anesthesia care unit, *PONV*, postoperative nausea and vomiting

## Discussion

In this randomized controlled study, laparoscopic-guided deep RSB significantly reduced postoperative opioid consumption within the first 24 h following elective laparoscopic cholecystectomy. The primary outcome, cumulative 24-h morphine consumption, was significantly lower in the deep RSB group. This opioid-sparing effect was consistent during the early postoperative period and was paralleled by lower pain scores at rest and during movement at 2, 6, and 12 h. However, the difference in pain intensity between groups reduced by 24 h. Although the incidence of rescue analgesia requirement and postoperative nausea and vomiting did not differ between groups, patients who received the block demonstrated higher quality-of-recovery scores at 24 h, as assessed by the QoR-15 questionnaire. These findings suggest that laparoscopic-guided deep RSB may provide effective early postoperative analgesia and may contribute to improved postoperative recovery.

Early postoperative pain following laparoscopic cholecystectomy is primarily related to trocar-site trauma and irritation of the parietal peritoneum resulting from pneumoperitoneum and surgical manipulation [1, 7]. Conventional abdominal wall techniques, including the TAP block and classical RSB, mainly target somatic afferents of the anterior abdominal wall and may have a limited effect on the parietal peritoneal component [19]. Also, a previous meta-analysis has shown that laparoscopic-assisted abdominal wall blocks provide comparable analgesic outcomes to ultrasound-guided techniques while offering the advantage of direct visualization of injectate spread, potentially improving procedural precision [20]. The present study targeted the preperitoneal plane, which may account for the more pronounced reduction in early postoperative opioid consumption observed in our cohort. In this context, the early reduction in both rest and movement-related pain observed in our study may be attributable to preperitoneal deposition of local anesthetic, which may extend analgesic coverage to the parietal peritoneal layer.

The strategy of targeting the preperitoneal space for postoperative analgesia has been previously described, and preperitoneal local anesthetic infiltration was initially reported in the context of laparoscopic hernia repair [8]. Subsequently, Fusco et al. described the deep RSB as an ultrasound-guided technique designed to deposit local anesthetic beneath the posterior rectus sheath, adjacent to the parietal peritoneum [9]. Case reports and technical descriptions suggested that this approach may extend analgesic distribution beyond that of the classical RSB, potentially affecting both the anterior cutaneous branches and the parietal peritoneal layer [13, 21]. Within this context, our study provides prospective randomized evidence demonstrating an opioid-sparing effect and improved early pain control. Importantly, in contrast to most previous reports describing ultrasound-guided deep RSB or preperitoneal approaches, our technique incorporated direct laparoscopic visualization of the injection plane. A recent case report has demonstrated the feasibility of laparoscopically guided deep rectus sheath and preperitoneal injection during laparoscopic cholecystectomy, confirming separation of the parietal peritoneum without perforation [11]. In the current study, the use of laparoscopic visualization allowed confirmation of the injection plane intraoperatively, which may increase the likelihood of accurate preperitoneal deposition. The anatomical basis and mechanism of action of the deep RSB rectus remain a subject of debate. A recent anatomical critique questioned whether injection within the “deep rectus sheath” represents a distinct compartment from an anterior transversalis fascia plane injection and suggested that direct blockade of the iliohypogastric or ilioinguinal nerves may be anatomically unlikely. The same critique emphasized that visceral analgesia through autonomic spread to the paravertebral or retroperitoneal space is unlikely with standard injectate volumes [22]. In contrast, the original proponents of the technique acknowledged that autonomic spread remains hypothetical and suggested that analgesia may be mediated through parietal peritoneal blockade and/or systemic absorption of local anesthetic [23]. Our findings align more closely with the parietal peritoneal hypothesis rather than a true visceral or autonomic mechanism. The significant reduction in early pain scores and opioid consumption, followed by convergence of pain intensity at 24 h, supports a predominantly early somatic or parietal effect rather than prolonged modulation of deeper visceral nociception. The anatomical relationship between the posterior rectus sheath, transversalis fascia, and parietal peritoneum is inherently complex and may differ above and below the arcuate line [22]. In this setting, the preperitoneal space can be narrow and sonographically subtle, which may pose challenges for consistent identification when the technique is performed under ultrasound guidance alone. In our study, the addition of laparoscopic visualization allowed direct confirmation of the injection plane and may therefore offer a practical technical advantage by improving consistency of preperitoneal deposition. Although anatomical and radiological studies are required to clarify the pattern of injectate spread and dermatomal distribution, our prospective randomized results support an association between preperitoneal deposition and enhanced early postoperative analgesia in laparoscopic cholecystectomy.

A notable methodological strength of the present study is the multimodal confirmation of block placement. In addition to intraoperative laparoscopic visualization of the injection plane, postoperative ultrasound verification with independent blinded assessors was used to objectively confirm injectate distribution. This strategy minimized the risk of misclassification of unsuccessful blocks as effective interventions and strengthened the internal validity of the evaluation of postoperative analgesic outcomes. The moderate interobserver agreement observed in ultrasound classification may reflect the inherent difficulty in visualizing the narrow preperitoneal space following pneumoperitoneum and surgical manipulation. Subtle fascial interfaces and redistribution of residual fluid may also contribute to variability in sonographic interpretation.

Effective postoperative pain management after laparoscopic cholecystectomy relies on a multimodal approach combining systemic non-opioid agents, regional anesthesia techniques, and selective opioid use. Current procedure-specific recommendations support paracetamol and non-steroidal anti-inflammatory drugs as baseline therapy, and also consider local anesthetic techniques such as port-site infiltration, intraperitoneal local anesthetic instillation, and selected regional blocks within a multimodal strategy. However, the routine use of the classical RSB remains controversial, largely due to limited or conflicting evidence [5]. In the present study, laparoscopically guided deep RSB was associated with reduced opioid consumption without an increase in complications, suggesting that it may represent a useful adjunct to standard care, especially in patients at higher risk for opioid-related adverse effects or in situations where improved early analgesia is a priority. The opioid-sparing effect observed in this study may be particularly relevant in patients at increased risk of opioid-related adverse events, including elderly patients or those with respiratory comorbidities. Compared with conventional port-site infiltration, preperitoneal targeting may provide broader early coverage of the midline and parietal peritoneal component of pain. From a practical perspective, the use of laparoscopic visualization during injection may improve accuracy but requires surgical collaboration and familiarity with the anatomical plane. Whether ultrasound-guided preperitoneal approach alone can consistently reproduce these effects remains uncertain and warrants further investigation.

Although postoperative opioid consumption was significantly reduced in the deep RSB group, this reduction was not accompanied by a statistically significant decrease in opioid-related adverse effects such as postoperative nausea and vomiting. In addition, while postoperative NRS pain scores were significantly lower at several early postoperative time points, the magnitude of these differences became smaller over time, particularly beyond the early postoperative period. Therefore, the clinical impact of the observed analgesic benefit should be interpreted cautiously. Nevertheless, even a modest opioid-sparing effect may still be clinically relevant within a multimodal analgesic strategy, particularly when accompanied by improved quality-of-recovery scores and without an increase in procedure-related complications.

There were several limitations to the current study. First, this was a single-center trial performed by a single surgical team, and all blocks were carried out by the same experienced surgeon. While this approach ensured technical consistency and reduced inter-operator variability, it may limit the generalizability and reproducibility of our findings across centers with different levels of surgical expertise or alternative technical approaches. Future multicenter studies involving multiple surgeons with varying levels of laparoscopic and regional anesthesia experience are needed to further clarify the external validity and broader applicability of this technique. Second, complete double blinding was not feasible due to the procedural characteristics of the intervention. While outcome assessors and data analysts remained blinded, the operating surgeon and intraoperative anesthesia team were aware of group allocation. Accordingly, the possibility of performance bias cannot be entirely excluded. However, predefined and strictly standardized anesthesia and analgesia protocols were implemented in both groups to minimize this risk. In addition, several patients were excluded after randomization because the primary outcome could not be reliably assessed due to technical reasons. Although these exclusions were predefined and related to outcome measurement rather than treatment response, they may introduce a potential risk of attrition bias. Third, the study did not include a sham-controlled intervention or an active comparator arm, such as trocar-site local anesthetic infiltration or classical rectus sheath block. Although a placebo intervention using saline injection was considered during study design, saline deposition within the preperitoneal plane itself was considered a potential source of tissue distension, peritoneal irritation, or procedure-related discomfort, which could introduce additional confounding effects. Furthermore, the primary objective of the study was to evaluate the incremental analgesic effect of laparoscopic-guided deep RSB when added to standard perioperative care rather than to compare different infiltration techniques. Fourth, although laparoscopic visualization and postoperative ultrasound examination were used to confirm preperitoneal distribution in the block group, we did not perform radiological contrast studies or dermatomal sensory mapping. Therefore, the exact pattern of spread and the specific neural structures affected by the block cannot be definitively determined. In addition, systemic plasma concentrations of local anesthetic were not measured, and a potential contribution of systemic absorption to the observed analgesic effect cannot be excluded. Finally, postoperative outcomes were assessed during the first 24 h only, and therefore longer-term analgesic outcomes remain uncertain. While early analgesia is particularly relevant after laparoscopic cholecystectomy, longer-term outcomes, including persistent postoperative pain, functional recovery beyond 24 h, and patient satisfaction after discharge, were not evaluated. In addition, only ASA I–II patients were included in the present study in order to maintain a relatively homogeneous study population and to minimize potential confounding effects of significant systemic comorbidities on postoperative pain perception, opioid consumption, and recovery outcomes in this initial randomized evaluation of the technique. Therefore, the applicability of these findings to patients with more advanced systemic comorbidities remains uncertain.

In conclusion, laparoscopically guided deep RSB was associated with reduced postoperative opioid consumption and improved early pain control within the first 24 h after elective laparoscopic cholecystectomy. The analgesic effect was most evident in the early postoperative period and was accompanied by better quality-of-recovery scores. These findings suggest that targeting the preperitoneal plane may enhance early multimodal analgesia; however, the results should be interpreted with caution due to the absence of long-term follow-up.

## Data Availability

The datasets generated and/or analyzed during the current study are available from the corresponding author upon reasonable request.
